# Does the DVR^®^ plate restore bony anatomy following distal radius fractures?

**DOI:** 10.1308/003588414X13824511650254

**Published:** 2014-01

**Authors:** S Patel, PB Menéndez, FS Hossain, HB Colaço, MH Lee, ED Sorene, EJ Taylor

**Affiliations:** University College London Hospitals NHS Foundation Trust,UK

**Keywords:** Radius, Internal fracture fixation, Cartilage fractures, Radiology information systems

## Abstract

**INTRODUCTION:**

Fractures of the distal radius are common. Malreduced fractures are associated with residual functional deficiency. There has been a trend over the last few years for using fixed angle volar locking plates to surgically stabilise this injury. Our unit uses the DVR^®^ plate (DePuy, Warsaw, IN, US). Nevertheless, it is unknown whether the normal bony anatomy is recreated or merely restored to acceptable limits with its usage. The aim of this study was to evaluate the reduction achieved compared with an uninjured population and pre-existing quoted ‘normal’ values. Furthermore, we wanted to identify the percentage of cases that were reduced to acceptable limits, and determine whether the grade of the surgeon and fracture type was a confounding influence on this reduction.

**METHODS:**

A retrospective review of the 3-month postoperative radiography of 48 eligible patients who underwent open reduction and internal fixation of a distal radius fracture with a DVR^®^ plate was undertaken.

**RESULTS:**

Volar tilt, radial length and inclination were different to quoted normal values (*p*<0.01). Despite this, these parameters fell within acceptable limits in 46 cases; this was not influenced by fracture type or grade of operating surgeon.

**CONCLUSIONS:**

The DVR^®^ plate restores the bony anatomy to within acceptable limits in the majority of patients who have sustained a fracture of the distal radius although of all parameters investigated, the widest variability is seen in volar tilt.

Fractures of the distal radius are common injuries with an estimated incidence of 71,000 cases in Britain every year.^1^ Patients of all ages can be affected but the elderly are more susceptible to injury.^2^ It has long been recognised that malreduced fractures are associated with poor long-term function.^3^ Consequently, there is an indication to reduce displaced fractures and surgically stabilise those that are unstable. The devices used for maintaining reduction surgically include percutaneous wires, external fixators, intramedullary nails and plates.

The early results of plate fixation were poor but improved dramatically following the introduction of precontoured locking plates. This has been associated with a rise in popularity with respect to usage. Recent randomised controlled trials have demonstrated better function in the early postoperative period with this fixation method than with other methods such as percutaneous pinning, external fixation and radial column plates although long-term function is comparable.^4,5^

The DVR^®^ plate (DePuy, Warsaw, IN, US) is a volar locking plate that was introduced in 2001. It has been shown to be biomechanically stable and possibly more so than other similar volar locking plates by other manufacturers.^6–8^ The DVR^®^ plate has undergone an evolution in design since first coming to the market with two rows of screw options now available (Fig 1) to improve subchondral support. Furthermore, the number of available sizes has been increased to seven so that it can be used in patients of variable bony anatomy and size.

**Figure 1:**
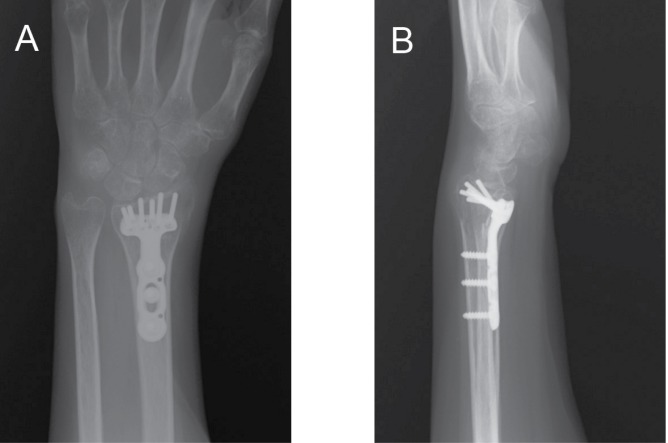
Standardised three-month postoperative posteroanterior (A) and lateral (B) radiography of a fractured distal radius that has been stabilised with a DVR^®^ plate

The biomechanical merits of plate osteosynthesis, complications and functional outcome are well documented. There is, however, a paucity of evidence as to what the expected radiographic parameters are following this fixation modality. Our institution always performs open reduction and internal fixation rather than closed reduction and percutaneous wire fixation for all adult patients presenting with an unstable fracture of the distal radius within two weeks of the injury. We have used the DVR^®^ plate with two rows of distal screw options since 2006 and we do not commonly image both wrists unless there is a suspicion of congenital abnormal anatomy.

‘Normal’ values for the volar tilt (VT), radial length (RL) and radial inclination (RI) of the distal radius were described originally in 1962 by Scheck as 11º, 12mm and 23º respectively.^9^ A subsequent pooled analysis of multiple studies has demonstrated the mean values to be 14.5º, 13.5mm and 25.4º respectively^10^ although it is the former set of values that continue to be quoted widely in the literature. The aims of this study were to: (1) evaluate the reduction at three months following surgery against that at the end of the procedure, against a group of uninjured wrists and against both sets of pre-existing quoted ‘normal’ values to determine whether these can be achieved using the DVR^®^ plate; (2) identify the percentage of cases that were reduced to acceptable limits; and (3) determine whether the grade of surgeon and fracture complexity were a confounding influence on this reduction.

## Methods

A retrospective analysis was undertaken of all patients with a fracture of the distal radius between 2008 and 2009 who underwent open reduction and internal fixation with a DVR^®^ plate. We identified 60 cases but excluded those patients whose fractures were stabilised with supplementary fixation (*n*=2), those lost to follow-up by the evaluation stage (*n*=3) and those who had a previous distal radius fracture (*n*=3) or inadequate radiography (*n*=4).

This left 48 cases in 48 patients (19 male, 29 female, mean age: 51.2 years, age range: 19–85 years). All fracture patterns were described according to the Arbeitsgemeinschaft für Osteosynthesefragen (AO) classification (type A: 13 cases, mean age: 38.3 years, age range: 19–79 years; type B: 9 cases, mean age: 48.2 years, age range: 21–72 years; type C: 26 cases, mean age: 59.1 years, age range: 21–85 years) and all procedures were performed within two weeks of injury by either a consultant (19 cases), a registrar with consultant supervision (4 cases) or a registrar independently (25 cases). The uninjured population with which these patients were compared as part of the analysis consisted of 48 wrists in 46 patients who presented to our clinic with a history of minor trauma to the upper limb and in whom no radiological abnormality was identified.

### Operative technique

All cases were operated on in a standardised manner under general anaesthesia and with a tourniquet inflated around the affected limb at 250mmHg for the duration of the operation. The distal radius was approached through the bed of the flexor carpi radialis tendon and the operation performed as per the recommended operative technique. The fracture was identified and reduced under fluoroscopy guidance. The plate was then positioned on the radial shaft, securing it initially using the central sliding hole. The decision to release the brachioradialis tendon to facilitate fracture reduction on to the plate was made by the operating surgeon. All available peg holes were then filled to maintain fracture reduction and prevent redisplacement. After securing the plate to the shaft with the final screws, the wound was closed and dressed in a bulky bandage.

### Postoperative protocol

Clinical and radiographic follow-up occurred at 2, 6 and 12 weeks following surgery, and thereafter based on clinical need. For weeks 2–6, patients were given a Futuro^®^ splint (3M, Bracknell, UK) for support that could be removed for exercises with our hand therapists. Discontinuation of the splint was advised routinely at week 6 and further supervised exercised was dependent on residual functional deficit.

### Radiographic assessment

Posteroanterior and lateral radiography was used to assess VT, RL and RI, with all values determined by two observers (SP and PBM). Intraoperative radiography was performed and further images were collected at least three months postoperatively to allow for any potential loss of fracture position. The first ten cases of three-month postoperative radiography were used for interobserver variability, showing good agreement (kappa = 0.91).

VT was defined as the angle created between the articular surface of the distal radius and a line perpendicular to the long axis of the radius as witnessed on lateral radiography. RL was defined as the distance between the tip of the radial styloid process and the distal articular surface of the ulna in a direction perpendicular to the long axis of the radius. RI was defined as the angle created between a line joining the tip of the radial styloid and the ulnar corner of the articular surface, and a line perpendicular to the long axis of the radius.

### Statistical analysis

Descriptive statistics were applied to describe the basic characteristics of the datasets. A two-sample unpaired t-test was used for comparing the uninjured population with the three-month postoperative radiography, a two-sample paired t-test for comparing the intraoperative radiography with the three-month postoperative radiography and a one-sample t-test for comparing the three-month postoperative radiographic parameters with normal values quoted in the literature to determine whether normal anatomy was recreated.

A one-way analysis of variance and covariance was used to determine whether these values differed depending on the grade and supervision of the operating surgeon, the type of the fracture as defined by the AO classification or the interaction between them. A chi-squared test was used to see whether complications varied according to the grade of the operating surgeon. All analyses were performed using the XLSTAT module (Addinsoft, Paris, France) for Excel^®^ (Microsoft, Redmond, WA, US), with statistical significance set at *p*<0.05.

## Results

### Comparison with normal values

Radiographic assessment at three months demonstrated that the mean VT achieved was 8.8º (standard deviation [SD]: 5.5º, range: -6–20º), the mean RL was 11.0mm (SD: 1.7mm, range: 7–15mm) and the mean RI was 21.0º (SD: 3.4º, range: 13–27º) (Table 1). Comparison of all values with normal values demonstrated that the difference was statistically significant for all parameters (Table 2).

**Table 1:** The number of patients and radiographic parameters (mean, range) by different grades of surgeon and fracture types

AO fracture type	Operating surgeon Consultant	Registrar with consultant supervision	Registrar	Overall
A	*n*=3VT: 6.0º (0–9º)RL: 11.3mm (10–14mm)RI: 23.3º (21–27º)	*n*=2VT: 3.5º (-6–13º)RL: 12.5mm (10–15mm)RI: 22.0º (21–23º)	*n*=8VT: 9.8º (4–14º)RL: 11.2mm (9–13mm)RI: 21.9º (16–26)	*n*=13VT: 8.0º (-6–14º)RL: 11.4mm (9–15mm)RI: 22.2º (16–27º)
B	*n*=2VT: 7.0º (5–9º)RL: 11.5mm (11–12mm)RI: 22.0º (22–22º)	*n*=1VT: 2.0º (N/A)RL: 11.0mm (N/A)RI: 15.0º (N/A)	*n*=6VT: 7.8º (-5–13º)RL: 10.7mm (7–15mm)RI: 22.0º (18–26º)	*n*=9VT: 7.0º (-5–13º)RL: 10.9mm (7–15mm)RI: 21.2º (15–26º)
C	*n*=14VT: 10.5º (0–20º)RL: 10.8mm (8–13mm)RI: 19.6º (16–24º)	*n*=1VT: 9.0º (N/A)RL: 14.0mm (N/A)RI: 24.0º (N/A)	*n*=11VT: 8.9º (-2–16º)RL: 10.6mm (9–13mm)RI: 20.9º (13–25º)	*n*=26VT: 9.8º (-2–20º)RL: 10.8mm (8–14mm)RI: 20.3º (13–25º)
Overall	*n*=19VT: 9.4º (0–20º)RL: 11.0mm (8–14mm)RI: 20.4º (16–27º)	*n*=4VT: 4.5º (-6–13º)RL: 12.5mm (10–15mm)RI: 20.8º (15–24º)	*n*=25VT: 8.9º (-5–16º)RL: 10.8mm (7–15mm)RI: 21.5º (13–26º)	*n*=48VT: 8.8º (-6–20º)RL: 11.0mm (7–15mm) RI: 21.0º (13–27º)

AO = Arbeitsgemeinschaft für Osteosynthesefragen; VT = volar tilt; RL = radial length; RI = radial inclination

**Table 2:** Comparison of obtained radiographic parameters with ‘normal’ values

Study	Volar tilt	*p*-value	Radial length	*p*-value	Radial inclination	*p*-value
Scheck, 1962^9^	11º	**0.007**	12mm	**<0.001**	23º	**<0.001**
Mann, 1992^10^	14.5º	**<0.001**	13.5mm	**<0.001**	25.4º	**<0.001**

### Comparison with uninjured wrists

The uninjured group was noted to have a mean VT of 8.5º (SD: 5.8º; range: -5–20º), a mean RL of 11.3mm (SD: 1.8mm, range: 7–16mm) and a mean RI of 24.3º (SD: 5.6º, range: 14–28º), with VT and RL remaining statistically different (*p*=0.02 and p=0.04 respectively); no difference was noted for RI (*p*=0.07).

### Comparison with intraoperative values

The mean intraoperative VT was 12.7º (SD: 0.8º, range: 8–17º), the mean RL was 13.1mm (SD: 2.7mm, range: 5–18mm) and the mean RI was 21.4º (SD: 3.6º, range: 13–28º). No statistical difference was noted when comparing these with the three-month postoperative values (*p*=0.87, p=0.91 and p=0.61 respectively).

### Comparison with acceptable values

Comparison with the radiographic criteria for acceptable healing of a distal radial fracture^11^ demonstrated that VT and RL were corrected for all patients but two patients had RIs of 13.0º and 14.4º, which thus approached an acceptable value but did not reach it (Table 3).

**Table 3:** The number and percentage of patients who had radiographic parameters within acceptable limits

	Acceptable measurement	Number of patients
Volar tilt	15º dorsal tilt – 20º volar tilt	48 (100%)
Radial length	>8.5mm	48 (100%)
Radial inclination	≥15º	46 (95.8%)

### Influence of fracture type and surgeon grade

Univariate and covariate analysis determined that neither fracture type, surgeon grade or the interaction between them affected VT (*p*=0.36, *p*=0.28 and *p*=0.67 respectively), RL (*p*=0.57, *p*=0.17 and *p*=0.71 respectively) or RI (*p*=0.25, *p*=0.63 and *p*=0.36 respectively).

### Complications

Follow-up of patients at 12 months determined that 9 patients had complications attributable to surgery, with 3 in the group where the consultant was the primary surgeon, 2 where the surgeon was a registrar operating under consultant supervision and 4 in the group where the registrar was operating independently; this was not statistically significant (*p*=0.32). Two patients had chronic regional pain syndrome: one suffered with a palsy of the sensory branch of the median nerve that had reversed by three months and one with a superficial wound infection that was treated successfully with oral antibiotics. Five patients required removal of the plate: two for poor plate positioning causing either joint or soft tissue impingement, one for placement of an intra-articular screw and two for fracture collapse with secondary joint impingement.

## Discussion

This study demonstrates that so-called normal values of distal radial anatomy are not replicated when using the DVR^®^ plate to treat unstable fractures of the distal radius with respect to VT, RL or RI. Despite this, the reduction achieved fell within acceptable limits in most cases irrespective of fracture complexity and the DVR^®^ plate can be used by surgeons of differing experience without compromising this. Of note, however, is that the complication rate in our series approached one in five cases, which reinforces the need for suitable training and patient selection.

This study was limited by its use of plain radiography as a measure of radiographic parameters. Although radiography was standardised, it is well recognised that rotation of the forearm, which could occur, may affect these parameters with pronation of 10º decreasing the apparent VT, RL and RI by 4.4º, 1.6mm and 2.8º respectively.^12^ There is consequently an argument for computed tomography assessment over plain radiography since it is more reliable for quantifying displacement.^13^ Unfortunately, our retrospective design did not allow for computed tomography in the present study. Furthermore, consideration would need to be given to the additional radiation exposure in any prospective study.

The second potential limitation of the study relates to the use of expected values against which radiographic parameters were compared rather than patients’ contralateral uninjured wrists. While it may be expected that a patient’s own anatomy would be a better comparison, the mean difference of 2.5º for VT, 1.5º for RI and 0.5mm for ulnar variance that has been shown to exist between the wrists of healthy subjects^14^ could limit its suitability. This is corroborated by Schuind *et al*, who compared the variability of right and left wrists on plain radiography with the variability of the distribution of those measurements from within the general population.^15^ It is noted that although the contralateral uninjured wrist should be used for assessing carpal measures, the normal side does not provide a better reference than normal values obtained from databases for VT, RI or ulnar variance.

The question of whether volar locking plates offer superior outcomes over other treatment options has been a recent topic for debate. Direct comparison with percutaneous wire fixation remains difficult as this method tends to be indicated only for extra-articular fractures whereas internal fixation methods such as volar locking plates can be used both for those that do and do not affect articular congruity.

In a specific cohort of patients older than 70 years, Arora *et al* demonstrated that radiographic parameters were significantly better in those treated with a locking plate than in those treated non-operatively.^16^ It is, however, worth noting that subjective and functional outcomes did not differ at a mean follow-up of 4 years and 7 months. When compared with external fixation, volar locking plates have been shown to have improved function at three months^4^ although this difference was no longer present subsequently, with either similar or better radiographic outcomes.^4,17^ These cumulative findings appear to support the use of volar locking plates for the treatment of fractures of the distal radius.

While our study demonstrated a disparity between normal values and the achieved reduction, the mean values fell within the accepted limits and this was not influenced by the grade of operating surgeon. However, since we now have discriminatory scoring and evaluation methods, we recommend these acceptable limits be investigated to see whether they still hold true. Although traditionally associated with outcome, there is recent evidence from a number of authors that in an elderly population aged >65–70 years, radiographic indices do not correlate with outcome.^16,18,19^ Taken together with the added cost effectiveness of percutaneous wires,^20^ this suggests that routine volar locking plate fixation in this group may not be justified.

The difficulty in discussing and evaluating volar locking plates comes from the wide variety of implants available, and to date, there are no reported human clinical studies comparing them. First generation DVR^®^ plates, which have a single row of distal screw holes, have been shown to be biomechanically superior to non-locking devices such as percutaneous wires^21^ or simple plates^6^ although they are comparable biomechanically with locking plates from other manufacturers under physiological loads^6,8,22–24^ and nonspanning external fixators.^25^ The addition of a second row of distal screw holes as found in the second generation DVR^®^ plates does not improve this.^7^ It is nevertheless worth noting that this design modification was to prevent subchondral collapse, which may be important clinically rather than biomechanically.

The clinical results of the DVR^®^ plate have been reported previously in 48 patients with AO type C fractures by Frattini *et* al.^26^ In this subgroup of patients, the radiographic outcomes were again within acceptable limits with a mean VT of 10.3º, a mean RL of 9.2mm and a mean RI of 23.2º. This is comparable with our patients with type C fractures. However, it is of note that we have shown acceptable radiographic parameters can be attained in the majority of patients irrespective of fracture type.

## Conclusions

We have shown that that the DVR^®^ plate, which is a fixed angle volar locking plate, is able to restore the bony anatomy to within acceptable limits in the majority of patients following an unstable fracture of the distal radius. The parameter that exhibits the widest variability is VT and while the rate of complication approached one in five cases, most were attributable to surgically related factors rather than failure of fixation, emphasising the need for suitable preoperative counselling of patients undergoing plate osteosynthesis of the distal radius irrespective of which implant is used.
